# Ankle synovial chondromatosis: Clinical, radiological, and surgical findings: A case report

**DOI:** 10.1016/j.radcr.2024.04.048

**Published:** 2024-05-17

**Authors:** Manizhe A. Kachuee, Iman Mohseni, Nikoo Emtiazi, Yasaman Sharifi

**Affiliations:** a Department of Radiology, Firoozgar Hospital, Iran University of Medical Sciences, Tehran, Iran; b Department of Pathology, Firoozgar Hospital, Iran University of Medical Sciences, Tehran, Iran; c Department of Radiology, School of Medicine, Iran University of Medical Sciences, Tehran, Iran

**Keywords:** Synovial chondromatosis, Ankle, Osteochondromatosis

## Abstract

Synovial chondromatosis is a rare benign condition defined by the presence of cartilaginous lesions in the synovium of joints, tendon sheaths, and bursae. It most typically affects large joints, such as the knee, hip, and shoulder, but it is also reported in smaller joints. Nonetheless, ankle involvement is relatively uncommon. A complete history and clinical, physical, and radiographic examinations are usually used to determine the diagnosis. Hence, we reported a case of a young patient with left ankle primary synovial chondromatosis who presented with a left ankle mass and chronic pain.

## Introduction

Synovial chondromatosis causes small cartilaginous masses to form as a result of metaplasia of synovial tissue. As these masses protrude from the synovium's internal surface, they become pediculized and eventually separate, resulting in free chondromas and loose bodies. They can ossify, therefore referred to as osteochondromatosis [1], [2], [3]. Synovial chondromatosis is most prevalent among men in their third to fourth decade of life, and it typically impacts large joints such as the knee and hip, with smaller joints being implicated less frequently [4], [5], [6]. Ankle involvement by osteochondromatosis is unusual [2]. Pain, swelling, and restricted motion are frequently reported by patients. Additionally, it can cause mechanical problems such as growing mass and stress on nearby structures (such as blood vessels or nerves) [3]. Herein, we present a case of primary synovial chondromatosis of the left ankle joint presenting as a palpable mass and secondary degenerative changes treated by surgical excision.

## Case report

**Fig. 1:**
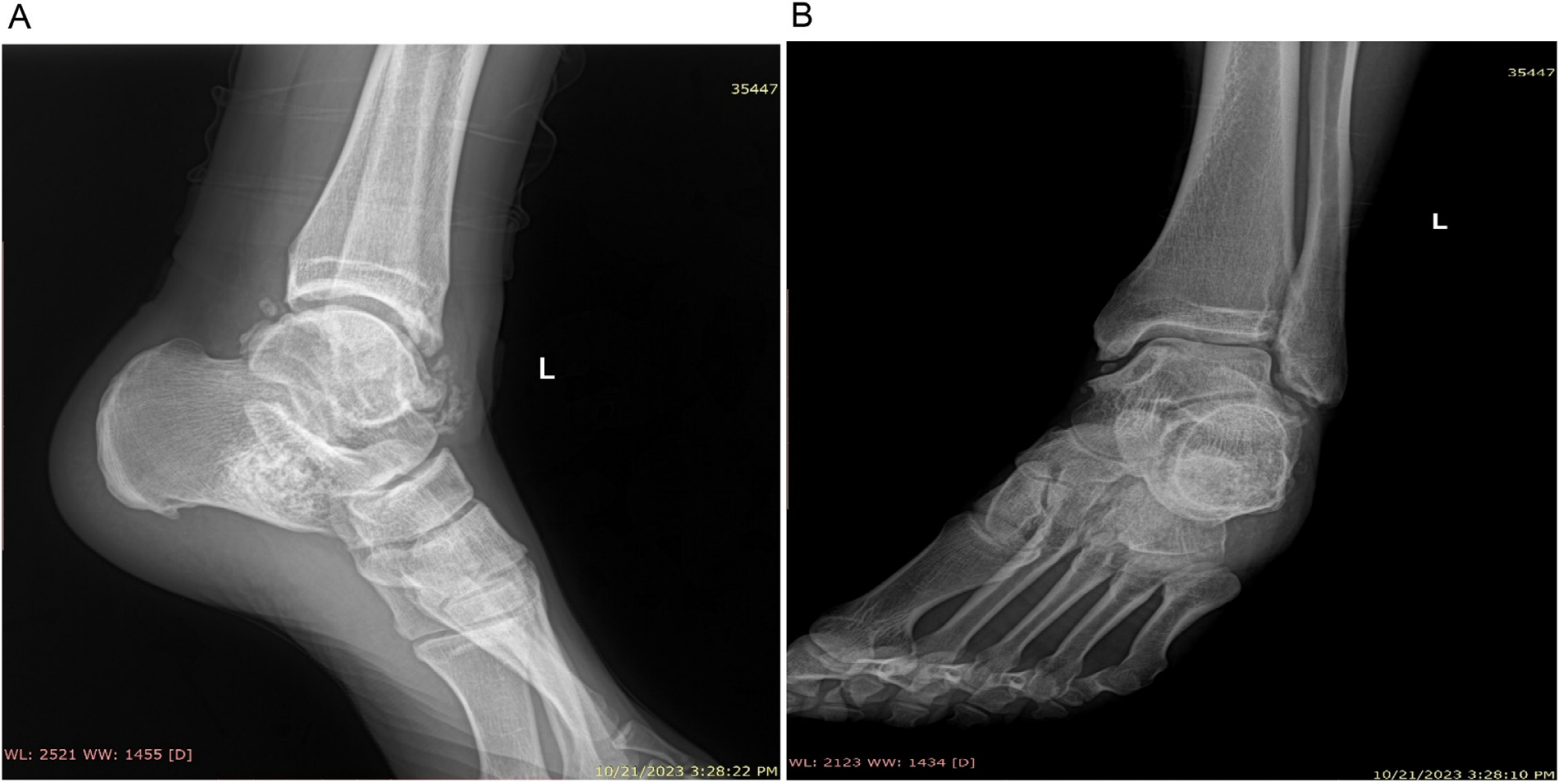
Preoperative (A) lateral and (B) mortise view radiographic images of the ankle joint illustrating several calcific loose bodies (Blue arrow) projecting posteriorly from the tibio-talar joint, as well as loose bodies anteriorly (Blue arrows).

A 33-year-old Iranian male presented to an orthopedic surgeon with a complaint of a palpable mass and pain in his left ankle. Walking wasn't restricted by pain, though he did experience severe, persistent pain over the dorsum of the left ankle. In the past 6 months, the mass had grown in size. The patient was otherwise healthy, and his past medical history and systems review were unremarkable. On examination, the patient's weight was within normal limits for his height. No lower limb alignment abnormalities or leg length inequalities were noted. There was no swelling or redness. He had difficulty heel-walking due to left ankle pain. Right ankle active and passive ranges of motion were reported as normal. The neurological examination of the lower limb was unremarkable. Orthopedic examination illustrated positive left ankle anterior drawer and synovial impingement maneuvers. The synovial chondromatosis was suspected and ankle radiographs were obtained. A radiograph showed several calcific loose bodies projecting posteriorly from the tibio-talar joint, as well as loose bodies anteriorly. The ankle mortise and subtalar joint spaces were well maintained. Several small osteophytes were noted at the lateral and posterior malleolus (Fig. 1).

**Fig. 2:**
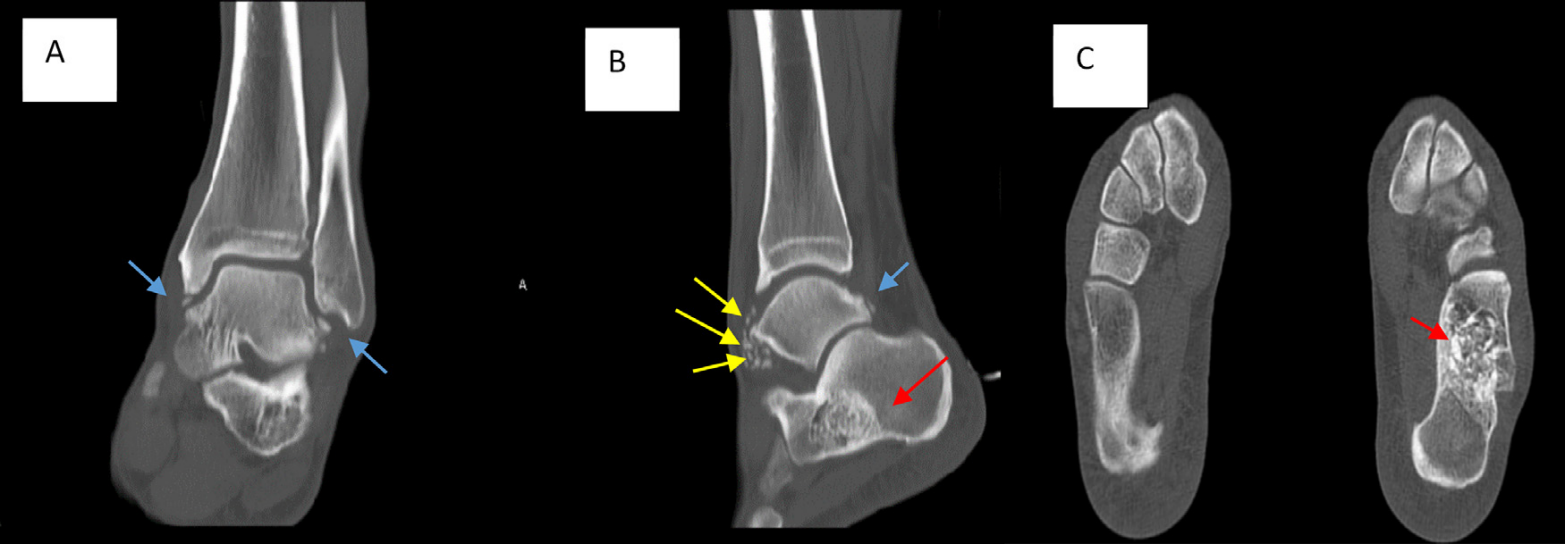
Computed tomography in A) Coronal View B) Sagittal View C) Axial View depicted that a lytic sclerotic lesion with a mixed density with a clear margin with a diameter of 57 mm is seen in the left calcaneus bone, inside which the appearance of the chondroid matrix is evident (Red arrow). Also, numerous uniform and ossified intra-articular loose bodies are seen in different parts of the left ankle, preferably in the anterior part of the talus and tibio-talar joint (Yellow arrows). Numerous osteophytes are seen in the distal tibia, talus, and fibula (Blue arrows).

The patient was a candidate for surgical mass removal. The computed tomography (CT) scan was also ordered for pre-surgical planning. In the CT scan, it was shown that a lytic sclerotic lesion with a mixed density and a clear margin with a diameter of 57 mm is seen in the left calcaneus bone, which seems to be a benign incidental finding. Also, numerous uniform and ossified intra-articular loose bodies are seen in different parts of the left ankle, preferably in the anterior part of the talus and tibio-talar joint. Numerous osteophytes are seen in the distal tibia, talus, and fibula. The collection of evidence suggested primary synovial chondromatosis with degenerative changes secondary to the intra-articular bodies (Fig. 2).

**Fig. 3:**
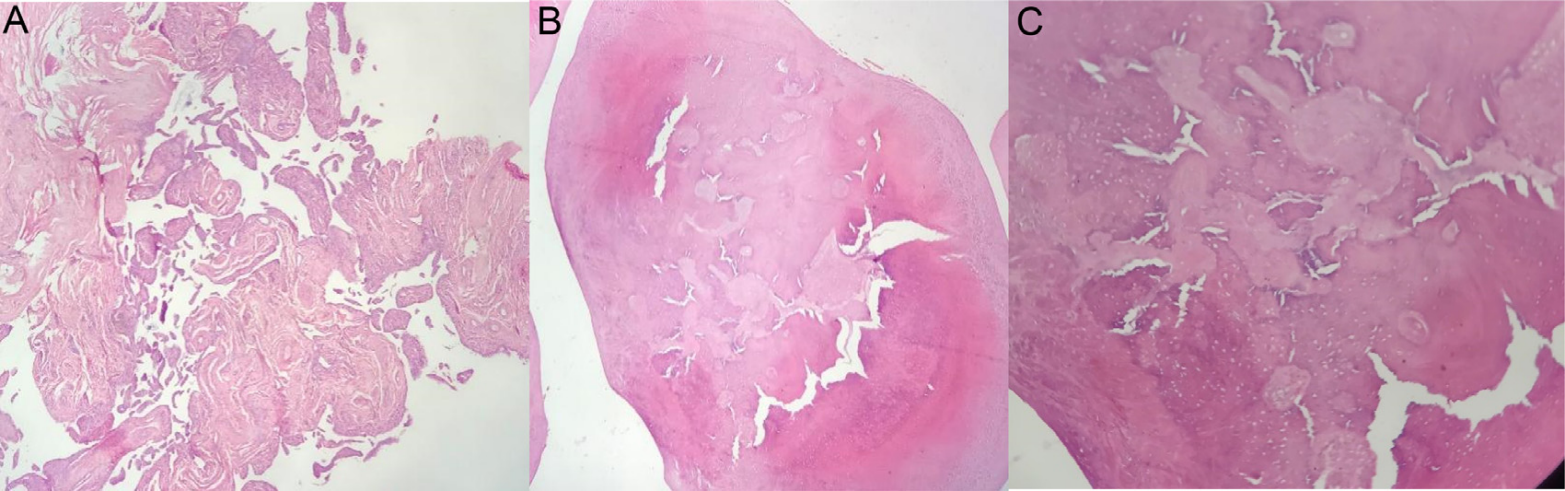
Pathology samples pictures were taken. (A) Synovial nodule embedded within the synovium which is covered by a layer of flattened synovial cells. (×4) (B) Nodules of metaplastic cartilage in synovium. (×4) (C) Nodules of cartilage in solid chondro myxoid background. (×10) All confirm the diagnosis of synovial chondromatosis.

The surgery has been done and mass excised. Multiple pearl-shaped lesions were sent for pathology. Pathology reported Synovial nodules embedded within the synovium, which is covered by a layer of flattened synovial cells, nodules of metaplastic cartilage in synovium, and also nodules of cartilage in a solid chondro myxoid background, all confirmed the diagnosis of synovial chondromatosis (Fig. 3).

## Discussion

Synovial chondromatosis is a benign, rare disease characterized by numerous pearl-like osteochondral loose bodies that are present intra- and extra-articularly [6], [7], [8]. Monoarticular involvement is most common, especially in large joints. Knee joints are involved in 60% to 70% of cases, followed by shoulders, elbows, and hips [6,9]. The condition of synovial chondromatosis of the foot and ankle is extremely unusual [6,10]. Galat et al. report only eight cases in the foot over 36 years, five of which were in the ankle. The same study reported only one case of an affected ankle among 25 patients with chondromatosis by Aguilera et al. [2,^10^,11].

Synovial chondromatosis is classified into primary and secondary types. Primary synovial chondromatosis was initially thought to be chondroid metaplasia in the synovium of a joint, resulting in the creation of several intraarticular chondral bodies. Secondary synovial chondromatosis is related to joint abnormalities, such as mechanical or arthritic diseases, which lead to intraarticular chondral bodies [12,13]. Secondary synovial chondrometaplasia's lesions were not aggressive. In contrast, primary synovial chondrometaplasia was aggressive and had a high recurrence rate. According to a review article, the incidence of both primary and secondary synovial chondromatosis is approximately 1.3% [14]. As far as we know, there is no published data on the exact prevalence of primary or secondary synovial chondromatosis, although secondary synovial chondromatosis is reported to be more common [15].

Our case study was categorized as primary synovial chondromatosis because there were no associated or stated joint problems, the patient had no history of trauma, his age for the degenerative joint disease was inappropriate, and the reported osteophytes and mild degenerative changes in the imaging studies were determined to be secondary to lose body formation in synovial chondromatosis.

The exact etiology of synovial chondromatosis remains unknown. Milgram et al. divided the disease process into 3 stages. The synovial lining undergoes cartilage metaplasia in the initial phase. The second phase is characterized by the separation of synovial nodules from the synovium and the appearance of loose bodies; this is the stage in which patients first become symptomatic. There is no sign of chondral metaplasia in the third phase, as the synovial mucosa has been destroyed. There are multiple loose bodies present within the joint cavity at this point, with no intrasynovial bodies visible, indicating that the synovium has decreased in activity. At this stage of the disease, nodules and loose bodies can be ossified or calcified [2,^6^,16].

Depending on the joint affected, the size, the loose bodies, and whether it's a primary or secondary chondromatosis, the clinical signs vary [2,6]. The symptoms of synovial chondromatosis typically include pain, swelling, stiffness, and/or a detectable mass, and most patients have a long clinical history before an accurate diagnosis is made [6].

Plain film radiographs are only useful in the third stage of the disease when calcification has taken place, according to Milgram's classification [16,17]. CT and MRI scans are types of advanced imaging that can be used to identify and locate lesions as well as help differentiate between other possible diagnoses. It's crucial to get a tissue biopsy when imaging is unable to reveal essential diagnostic details. A synovial tissue sample is used histologically to make a final diagnosis. Specific differential diagnoses can also be ruled out with the assistance of blood testing and arthritic profiles [17].

Osteochondritis dissecans, synovial vascular malformation, pigmented villonodular synovitis, chondrosarcoma, injury-related soft-tissue calcification, and lipoma arborescence with osseous metaplasia are all possible differential diagnoses [17], [18], [19].

Open or arthroscopic surgical excision is the recommended treatment. When active synovitis, generally stage 1 or stage 2, is present, a synovectomy is required. Since the majority of patients arrive at the late stage, when active synovitis is no longer present, a synovectomy is not required [20]. Recurrence happens in between 3% to 23% of scenarios and is thought to occur following synovectomy with active synovium remaining or in the presence of the stimulus that caused the metaplasia [21]. Our patient underwent open surgical excision and loose body removal and was without recurrence at the last follow-up.

## Conclusion

In the current study, we present a case of left ankle primary diffuse synovial chondromatosis. This rare, unique benign synovial neoplasm has clinical, radiological, and pathological features that are straightforward to recognize. Primary synovial chondromatosis is effectively treated with arthroscopic or open, loose-body excision. The prognosis is extremely favorable.

## Patient consent

Written informed consent was obtained from the patient for publication of this case report and accompanying images. A copy of the written consent is available for review by the Editor-in-Chief of this journal on request.

## References

[1] Moorthy V., Tay K.S., Koo K. (2020). Arthroscopic treatment of primary synovial chondromatosis of the ankle: a case report and review of literature. J Orthopaed Case Rep.

[2] Diop B., Daffe M., Sarr N., Faye I., Ndoye A.Y., Sané J.C. (2022). Primary synovial chondromatosis of the ankle: A case report. Int J Surg Open.

[3] Adelani M.A., Wupperman R.M., Holt G.E. (2008). Benign synovial disorders. JAAOS-J Am Acad Orthopaed Surgeons.

[4] Chen C.-Y., A.C.-Y. Chen, Chang Y.-H., Fu T.-S., Lee M.S. (2003). Synovial chondromatosis of the hip: management with arthroscope-assisted synovectomy and removal of loose bodies: report of two cases. Chang Gung Med J.

[5] Shpitzer T., Ganel A., Engelberg S. (1990). Surgery for synovial chondromatosis: 26 cases followed up for 6 years. Acta Orthop Scand.

[6] Sedeek S.M., Choudry Q., Garg S. (2015). Synovial chondromatosis of the ankle joint: clinical, radiological, and intraoperative findings. Case Rep Orthoped.

[7] Hocking R., Negrine J. (2003). Primary synovial chondromatosis of the subtalar joint affecting two brothers. Foot Ankle Int.

[8] Kistler W. (1991). Synovial chondromatosis of the knee joint: a rarity during childhood. Eur J Pediatr Surg.

[9] Walling A.K., Gasser S.I. (1994). Soft-tissue and bone tumors about the foot and ankle. Clin Sports Med.

[10] Galat D.D., Ackerman D.B., Spoon D., Turner N.S., Shives T.C. (2008). Synovial chondromatosis of the foot and ankle. Foot Ankle Int.

[11] Wiedemann N.A., Friederichs J., Richter U., Bühren V. (2011). Secondary synovial chondromatosis of the ankle joint. Der Orthopäde.

[12] Murphey M.D., Vidal J.A., Fanburg-Smith J.C., Gajewski D.A. (2007). Imaging of synovial chondromatosis with radiologic-pathologic correlation. Radiographics.

[13] Villacin A.B., Brigham L.N., Bullough P.G. (1979). Primary and secondary synovial chondrometaplasia: histopathologic and clinicoradiologic differences. Hum Pathol.

[14] Bergovec M., Kubat O., Smerdelj M., Seiwerth S., Bonevski A., Orlic D. (2015). Epidemiology of musculoskeletal tumors in a national referral orthopedic department. A study of 3482 cases. Cancer Epidemiol.

[15] Ko E., Mortimer E., Fraire A.E. (2004). Extraarticular synovial chondromatosis: review of epidemiology, imaging studies, microscopy and pathogenesis, with a report of an additional case in a child. Int J Surg Pathol.

[16] Milgram J., Addison R. (1976). Synovial osteochondromatosis of the knee. Chondromatous recurrence with possible chondrosarcomatous degeneration. JBJS.

[17] Shearer H., Stern P., Brubacher A., Pringle T. (2007). A case report of bilateral synovial chondromatosis of the ankle. Chiropract Osteop.

[18] Gerard V.Y., Zema R.L., Johnson R.W. (2002). Synovial osteochondromatosis: a case report and review of the literature. J Am Podiatr Med Assoc.

[19] Jeon I.-H., Ihn J.-C., Kyung H.-S. (2004). Recurrence of synovial chondromatosis of the glenohumeral joint after arthroscopic treatment. Arthroscop: J Arthroscop Relat Surg.

[20] Dworak D.P., McGuire M.H. (2011). Primary synovial osteochondromatosis in the ankle: a case report. Am J Orthop (Belle Mead NJ).

[21] Krebs V.E. (2003). The role of hip arthroscopy in the treatment of synovial disorders and loose bodies. Clin Orthopaed Related Res®.

